# Transcriptional Analysis of Total CD8^+^ T Cells and CD8^+^CD45RA^-^ Memory T Cells From Young and Old Healthy Blood Donors

**DOI:** 10.3389/fimmu.2022.806906

**Published:** 2022-01-27

**Authors:** Georgiana Toma, Ioana Maria Lemnian, Eliza Karapetian, Ivo Grosse, Barbara Seliger

**Affiliations:** ^1^Institute for Medical Immunology, Martin-Luther University Halle-Wittenberg, Halle, Germany; ^2^Institute for Computer Science, Martin-Luther University Halle-Wittenberg, Halle, Germany; ^3^Institute for Human Genetics, Martin-Luther University Halle-Wittenberg, Halle, Germany; ^4^German Centre for Integrative Biodiversity Research (iDiv) Halle-Jena-Leipzig, Leipzig, Germany; ^5^Department for Therapeutics, Fraunhofer Institute for Cell Therapy and Immunology, Leipzig, Germany

**Keywords:** aging, CD8+, T cells, RNA sequencing, memory T cells, JAK/STAT pathway, GO terms

## Abstract

Memory CD8^+^ T cells accumulate with aging, while the naïve T cell compartment decreases, leading to an increased susceptibility to infections and a decreased vaccine efficiency. To get deeper insights into the underlying mechanisms, this study aims to determine the age-dependent expression profile of total versus memory CD8^+^ T cells from young and old donors. Total CD8^+^ and CD8^+^CD45RA^-^ memory T cells isolated from young (<30 years) and old (>60 years) donors were stimulated with anti-CD3 and anti-CD28 antibodies for 48h before analyzing the cytokine secretion and activation markers by flow cytometry and changes in the expression profiles using RNA sequencing. Gene ontology (GO) term enrichment analyses were performed for up-regulated and uniquely expressed transcripts identified in the T cell populations of both age groups. Total and memory CD8^+^ T cells from old donors expressed significantly higher CD25 levels and have an increased cytokine secretion. While approximately 1,500 up-regulated transcripts were identified in all groups, CD8^+^CD45RA^-^ memory T cells of old donors had approximately 500 more uniquely expressed transcripts. Four GO terms related to the JAK-STAT pathway were identified for up-regulated transcripts in the total CD8^+^ T cells of old donors, whereas CD8^+^CD45RA^-^ memory T cells GO terms related to adjacent pathways, like JNK and MAPK/ERK, were found. Additionally, the unique transcripts of CD8^+^CD45RA^-^ memory T cells of old donors were related to the JNK, MAPK and IL-12 pathways. For both T cell populations of the old donors, cytokine and JAK-STAT pathway transcripts were up-regulated. Thus, an age-dependent effect was observed on the transcriptomes of total and memory CD8^+^ T cells. The CD8^+^ CD45RA^-^ memory T cells from old donors maintained the increased cytokine secretion of the total CD8^+^ T cell population and the increased JAK-STAT pathway transcripts, which have an impact on inflammation and senescence.

## Introduction

A consequence of aging is the reduced efficacy of immune responses and the depletion of the naïve T cell pool accompanied by the increase of memory T cell subpopulations during aging is a well-documented phenomenon (1–7). Compared to CD8^+^CD45RA^+^ naïve T cells, memory CD8^+^CD45RO^+^ T cells were exposed to an antigen and do not require a co-stimulatory signal for activation (8). Hence, memory CD8^+^ T cells cannot respond to newly encountered antigens, thereby contributing to the increased susceptibility to infections (9–13) as well as a reduced efficiency of vaccines in old individuals (14).

Furthermore, HLA-DRB1^+^ memory CD8^+^ T cells, which have regulatory properties (15, 16), accumulate with aging (17) and have also been used for determining the metastatic status of breast cancer patients (18). Since memory CD8^+^ T cells are easily activated and produce more pro-inflammatory factors than unprimed naïve T cells (19) they participate together with increased visceral fat, increased gut permeability and chronic viral infections (20)in creating a low level of chronic inflammation which was observed in old individuals. This implies an increase in the circulation of pro-inflammatory factors, such as interleukin-6 (IL-6), tumor necrosis factor-alpha (TNF-α) and interferon-gamma (IFN-γ) (21, 22), while circulating anti-inflammatory factors were decreased or varied within old individuals (22, 23).

So far, the increase of circulatory memory CD8^+^ T cells in peripheral blood has been extensively analyzed in old individuals (1–7) using high-throughput assays such as mass cytometry, which identified CD8^+^ T cell subpopulations expressing CD5, CCR2, CCR4, CXCR6 or CXCR4 to accumulate in old individuals (24).

In contrast, only a few studies compared CD8^+^ T cell subpopulations from young and old individuals. One marker for aging is the loss of CD28 on T cells, which provides the secondary costimulatory signal for naïve T cells, essential for their activation (25). High-resolution microarrays CD8^+^CD28^-^ T cells from both young and old individuals showed similar gene expression patterns, while CD8^+^CD28^+^ T cells from old individuals exhibit a gene expression pattern comparable to that of CD8^+^CD28^-^ T cells rather than that of CD8^+^CD28^+^ T cells from young individuals (26). This technology was also employed for the analysis of gene expression changes during T cell activation and the kinetics of specific gene clusters (27). In addition, the chromatin accessibility of different CD8^+^ T cell subpopulations from different age groups demonstrated a progression toward an effector-like chromatin accessibility pattern in the naïve and central memory CD8^+^ T cell subpopulations from old individuals (28).

So far, gene expression patterns during T cell activation and distinct CD8^+^ T cell subpopulations were compared between young and old individuals, but the expression profiles of memory CD8^+^ T cells from young and old individuals have not yet been compared after T cell activation.

RNA sequencing-based approaches have been used for the analysis of gene expression patterns and differentiation of CD8^+^ T cells after vaccination or after response to immunotherapy (29, 30). This high-throughput method was also applied for comparing different routes of administration for personalized cancer vaccines and the functional activity of neo-antigen specific CD8^+^ T cells (31). Furthermore, it could be demonstrated that an aged host environment leads to the development of granzyme K-expressing CD8^+^ T cells, which have a comparable transcriptional signature to exhausted CD8^+^ T cells (32).

It is noteworthy that many cytokines signal *via* the JAK/STAT pathway. Hence, the JAK/STAT signaling pathway was shown to contribute to the regulation of differentiation, proliferation, migration and cytotoxicity of T cells in response to cytokines (33). Many of these cytokines are crucial for T cell function, including IL-2 (34), IL-4 (35), IL-12, and IFN-γ (36, 37). To better understand the underlying cellular mechanisms of impaired T cell functionality occurring during aging, the transcriptional profiles of total CD8^+^ and CD8^+^CD45RA^-^ memory T cells of young and old healthy blood donors were compared in an exploratory study using high throughput RNA sequencing.

## Materials and Methods

### Biological Material

Peripheral blood mononuclear cells (PBMC) were purified from blood with the gradient method (Bicoll, 1.077 g/ml, Biochrom, Berlin, Germany). The healthy human blood donors were separated into young (23-30 years) and old (60-68 years) donors (**Supplementary Table 1**). These two age groups were selected based on published evidence, which shows that the two selected ages are distinct enough for the current study (38). The blood was provided by the blood bank of the University Hospital in Halle (Saale), Germany, upon donor consent. In total, blood from 32 healthy donors (equal numbers of women and men) was analyzed. Due to the limited numbers of cells that can be isolated, these donors were split into two cohorts for each one cell population,

### Isolation and Activation of T Cells

The total CD8^+^ T cells were magnetically sorted from PBMC using CD8^+^ beads (Miltenyi, Bergisch Gladbach, Germany) according to the manufacturer’s protocol. For the isolation of CD8^+^CD45^-^ memory T cells, the whole CD8^+^ T cell population was sorted first by magnetic positive purification using a MultiSort Kit (Miltenyi, Bergisch Gladbach, Germany). Then the CD8^+^CD45RA^-^ memory T cells were purified using a memory CD8^+^ kit (Miltenyi, Bergisch Gladbach, Germany) according to the manufacturers’ recommendations. The gating strategy is presented in the **Supplementary Figure 1**. Only preparations with a purity of CD3^+^CD8^+^ or CD8^+^CD45RA^-^ T cells of >95% were used. All cells were cultured in X Vivo-15 medium (Lonza, Verviers, Belgium) supplemented with 1% penicillin-streptomycin mixture (Sigma-Aldrich Chemie GmbH, Taufkirchen, Germany) and 1% L-glutamine (Lonza). The T cells were seeded at a density of 10^6^ cells/ml, activated with 2.5 µg/ml plated anti-CD3 monoclonal antibody (mAb; clone OKT3; Thermo Fisher Scientific, Waltham, MA, U.S.A.) and 1 µg/ml soluble anti-CD28 mAb (clone REA1047; BD Biosciences, San Jose, CA, U.S.A.) and then cultured for 48 hours. The anti-CD3 mAb was plated for at least 16 hours before each assay at 4°C in sterile phosphate-buffered saline (PBS). All flow cytometric data were acquired using a BD LSRFortessa flow cytometer with the BD FACSDiva software (both from BD Biosciences).

### Assessment of T Cell Activation

To assess the activation levels (39), total CD8^+^ T cells and CD8^+^CD45RA^-^ memory T cells were activated for 48 hours and approximately 10^5^ cells were washed once with ice-cold PBS supplemented with 2mM ethylenediaminetetraacetic acid (Sigma-Aldrich GmbH) and 0.5% (v/v) fetal calf serum (PAN Biotech, Aidenbach, Germany), before they were stained with anti-CD69 FITC, anti-CD25 PE (BD Biosciences) and anti-CD71 APC-H7 (Biolegend, San Diego, CA, U.S.A.) mAbs for 10 min in the dark on ice. The stained cells were subjected to flow cytometric analysis and the percentages of cells expressing each activation surface marker were determined by gating the positive cells in the histogram of each fluorophore.

In addition, the cell supernatant of each preparation was collected after 48 hours, aliquoted and stored at -20°C until use. The concentration of cytokines was determined using BD Cytometric Bead Array Flex Sets (BD Bioscience) containing IFN-γ, TNF-α, IL-3, IL-4, IL-5, IL-6, IL-10, granulocyte-macrophage colony-stimulating factor (GM-CSF), granzyme A and granzyme B according to the manufacturer’s specifications. The cytokine secretion data were analyzed using the FCAP Array software (BD Biosciences). The results are presented as pg/ml or ng/ml.

### Total RNA Isolation

After 48 hours of activation, T cells were pelleted and washed once with ice-cold PBS, before the cell pellet was treated with the lysis buffer provided in a NucleoSpin RNA Isolation Kit (Macherey-Nagel, Dueren, Germany) supplemented with β-mercaptoethanol (AppliChem GmbH) as indicated in the kit manual and either stored in a -80°C freezer or directly used for RNA isolation. Total RNA was isolated from the cell lysate using the NucleoSpin RNA Isolation Kit according to the manufacturer’s instructions. RNA concentrations were determined with an Infinite 200 PRO Microplate Reader (Tecan Group Ltd., Mannedorf Switzerland) and NanoQuant Plate™ (Atlantic Lab Equipment, Beverly, MA, U.S.A.).

### Relative Gene Expression

Total RNA was subjected to quantitative PCR (qPCR). For each sample, complementary DNA (cDNA) was synthesized from 500 ng of total RNA using a RevertAid First Strand cDNA synthesis kit (Thermo Fisher Scientific). qPCR reactions were performed using the 2 X SYBR Green qPCR Master Mix (Promega, Madison, WI, USA) according to the manufacturer’s recommendations. The primers used are listed in **Supplementary Table 2**. qPCR data are presented as a fold change calculated using the quantification cycle (Cq) according to the formula ΔΔCq =(CqHK-CqGOI)young-(CqHK-CqGOI)old (40). CqHK represents the mean of the Cq values for the housekeeping genes and CqGOI represents the Cq value of the gene of interest for the indicated age group. The genes of the TATA-binding box protein (TBP) and the succinyl dehydrogenase subunit A (SDHA) were used as housekeeping genes.

### RNA Sequencing and RNA-Seq Data Analysis

**Figure 1:**
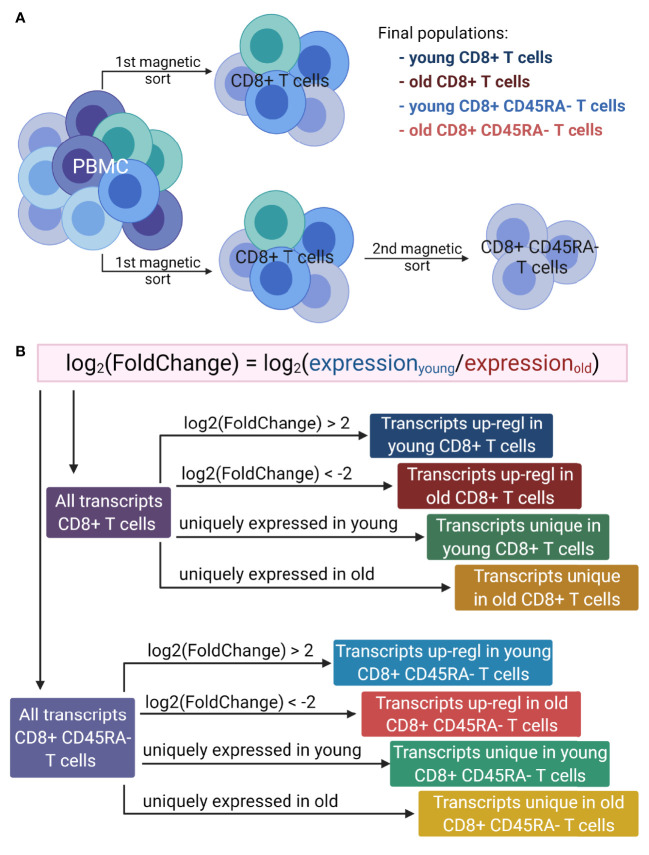
Overview of the workflow. **(A)**. Generation of T cell populations used for RNA sequencing. PBMC were isolated from multiple donors of two age groups: young (<30 years) and old (>60 years). The total CD8^+^ T cells were magnetically sorted from the PBMC directly, and to isolate the CD8^+^CD45RA^-^ memory T cells, the total CD8^+^ T cells were magnetically sorted from the PBMC and then the memory T cells were isolated from the total CD8^+^ T cells. This protocol was applied for both age groups. **(B)**. Lists of transcripts used for GO term enrichment. For both T cell populations, the log_2_(FoldChange) of gene expression was calculated as the expression of T cells of young versus that of the old donors. The transcripts up-regulated in either young or old donors were determined based on the log_2_(FoldChange) value. Two additional lists were generated for each cell population, based on the absence of expression for one of the age groups. Up-regl = up-regulated. The figure was prepared with Biorender.com.

**Figure 2:**
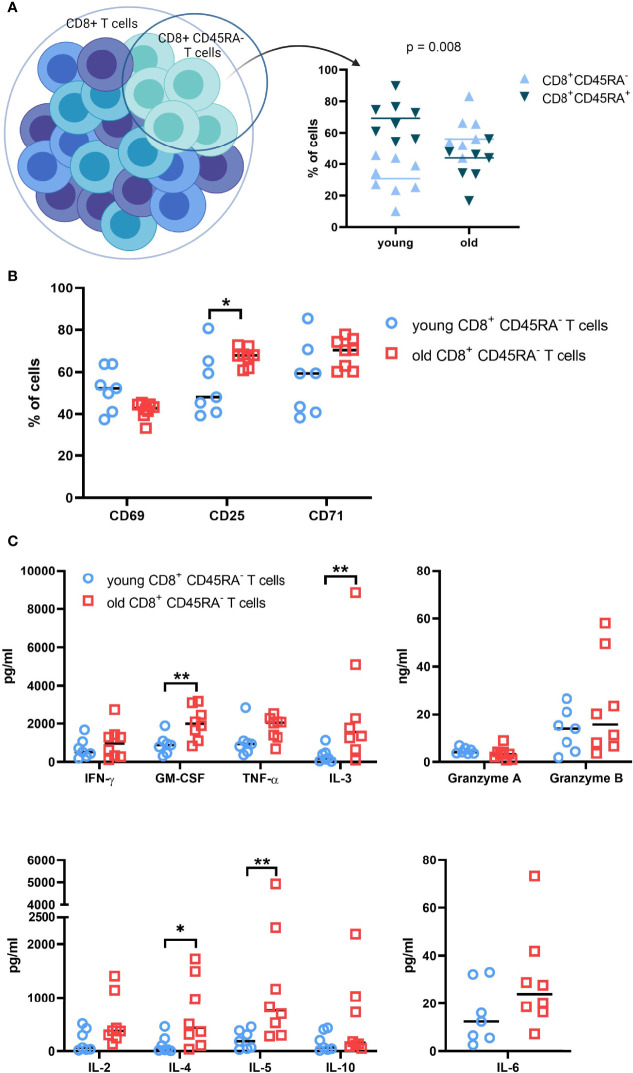
Functional analysis of T cell subpopulations subjected to RNA sequencing. **(A)**. Composition of the total CD8^+^ T cell populations of young and old donors. Pre-sort distribution of the CD8^+^ CD45RA^-^ memory T cell subpopulation within the total CD8^+^ T cell population preparations were determined with flow cytometry. N = 8. **(B)**. Expression of activation markers on CD8^+^CD45RA^-^ memory T cell subpopulations. The surface expression of common activation markers was measured after 48h. N = 8. * = p value < 0.05, ** = p value < 0.01. **(C)**. Soluble-protein secretion patterns of CD8^+^ CD45RA^-^ T cells from young and old donors. Soluble proteins were measured in the supernatant after 2 days of culture with a CBA Flex Set from BD Biosciences. The kit contains granzymes A and B, IFN-γ, TNF-α, GM-CSF, IL-10, IL-6, IL-5, IL-4, IL-3, and IL-2. N=8; * = p value < 0.05, ** = p value < 0.01.

For RNA sequencing, total RNA from eight donors for each T cell subpopulation was prepared (**Figure 1A**) and 2 µg/sample was processed by Novogene (Hong Kong, China) in two batches using the NEBNext^®^ UltraTM RNA Library Prep Kit for Illumina^®^ (NEB, USA) and the HiSeq-PE150 platform. The quality of the sequencing data was assessed using FastQC^1^ and MultiQC (41). Adapter sequences and quality nucleotides were trimmed using Trimmomatic (42). The quantification of the gene expression at the transcript level was performed by computing a pseudo-alignment to the human reference genome (GRCh38) using Kallisto (43). Additional bioinformatics analyses were performed using the programming language R^2^. The R package tximport (44) was used for importing the estimated read counts and for summarizing the transcript abundances. For the CD8^+^CD45RA^-^ memory T cells, one donor was excluded from further analysis for technical reasons.

Differentially expressed transcripts for the two comparisons of (i) total CD8^+^ T cells from young versus those from old donors and (ii) CD8^+^CD45RA^-^ memory T cells from young versus those from old donors were calculated using the DESeq2 package (45). Lowly abundant transcripts with less than 10 reads in total were removed from the analysis. For each transcript, DESeq2 fits a generalized linear model indicating the overall expression level of the transcript and returning the log_2_(FoldChange) between samples from young and old donors. The samples of old donors were used as controls, therefore, a negative value of the log_2_(FoldChange) represents an up-regulation in old donors (or a down-regulation in young donors), whereas a positive value an up-regulation in young donors (or a down-regulation in old donors). P values were calculated using the Wald test and then adjusted for multiple testing using the Benjamini-Hochberg correction. The variation between the samples of each CD8^+^ T cell population was visualized using the principal component analysis (PCA).

The final list of transcripts was divided into (i) uniquely expressed transcripts, which were expressed by only one age group and (ii) up-regulated transcripts, setting a threshold for the log_2_(FoldChange) value below -2 or above +2. Transcripts with a log_2_(FoldChange) value over 2 were considered up-regulated in young donors or down-regulated in old donors and transcripts with a log_2_(FoldChange) below -2 were designated as down-regulated in young or up-regulated in old donors.

### Gene Ontology (GO) Term Enrichment Analysis

GO term enrichment analysis was performed on eight lists of transcripts (**Figure 1B**) containing (i) up-regulated transcripts in young donors for the total CD8^+^ T cells, (ii) up-regulated transcripts in old donors for the total CD8^+^ T cells, (iii) uniquely expressed transcripts in young donors for the total CD8^+^ T cells, (iv) uniquely expressed transcripts in old donors for the total CD8^+^ T cells, (v) up-regulated transcripts in young donors for the CD8^+^CD45RA^-^ memory T cells, (vi) up-regulated transcripts in old donors for the CD8^+^CD45RA^-^ memory T cells, (vii) uniquely expressed transcripts in young donors for the CD8^+^CD45RA^-^ memory T cells and (viii) uniquely expressed transcripts in old donors for the CD8^+^CD45RA^-^ memory T cells. Up-regulated transcripts were ranked according to their adjusted p values, and uniquely expressed transcripts were ranked according to the average of the normalized count values of all samples.

GO term enrichment analysis was performed using the gene ontology enrichment analysis and visualization tool^3^ (GOrilla) with the “one ranked list” option, which identifies those transcripts at the top of a ranked list that most likely participates in shared functions with the minimal hypergeometric test (46). The overlap between various lists of genes and transcripts was assessed using the interactive tool for comparing lists with Venn`s diagrams Venny 2.1.0^4^. The visualization of the overlap was done with Biorender^5^.

## Results

### Increased Activation of Both Total CD8^+^ T Cells and CD8^+^ CD45RA^-^ Memory T Cells of Old Donors

The distribution of CD8^+^CD45RA^-^ memory T cells in the preparations of total CD8^+^ T cells was determined prior to sorting in order to examine the differences in the T cell populations between young and old donors. The CD8^+^CD45RA^-^ memory T cells represent on average 30% and 60% of the total CD8^+^ T cell population for the young and old donors, respectively (**Figure 2A**).

After 48h of culture, the cell surface expression of the activation markers CD25, CD69, and CD71 was measured for the CD8^+^CD45RA^-^ memory T cells. The expression of CD69 was very heterogeneous for the young donor groups, but stable within the old donor groups with no significant difference between the age groups (**Figure 2B**). In contrast, CD25 expression was increased in CD8^+^CD45RA^-^ memory T cells compared to the total CD8^+^ T cells (data not shown). The young CD8^+^CD45RA^-^ T cells exhibited a more heterogeneous and reduced expression of CD25 compared to that of old donors. CD71 expression was comparable to CD25 expression, but with an increased heterogeneity in the old donor groups.

In addition, the secretion pattern for the CD8^+^CD45RA^-^ memory T cells had a heterogeneous distribution. Concentrations of TNF-α, IL-6, and granzyme A were comparable between young and old donors, while concentrations of GM-CSF, IL-3, IL-4 and IL-5 were significantly higher in old donors than in young donors. Secretion of granzyme B, Il-2 and Il-10 was marginally increased for the old donors. This expression pattern was also confirmed by qPCR (**Supplementary Figure 2A**).

### Distinct Transcriptome Profiles of Total CD8^+^ T Cells and CD8^+^CD45RA^-^ Memory T Cells in Young and Old Donors

To determine differences in the expression profiles, total RNA from CD8^+^ and CD8^+^CD45RA^-^ memory T cells of both young and old donors was subjected to RNA sequencing. In total, 32 samples were sequenced at a depth of 30 million paired-end reads on average with eight biological replicates in each T cell population and age group. A total of 1,443 and 1,400 transcripts were up-regulated in young and old donors, respectively, for the total CD8^+^ T cells and 1,598 and 1,579 transcripts were up-regulated in young and old donors, respectively, for the CD8^+^CD45RA^-^ memory T cell population. Additionally, 2,757 and 2,777 transcripts were uniquely expressed in young and old donors, respectively, for the total CD8^+^ T cells, and 2,510 and 3,302 transcripts were unique in young and old donors, respectively, for the CD8^+^CD45RA^-^ memory T cell population.

**Figure 3:**
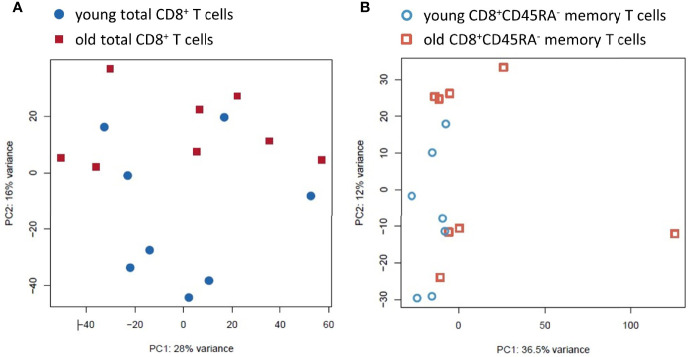
Principal Component Analysis of the transcriptomic profiles of the total CD8^+^ T cell population and the CD8^+^CD45RA^-^ memory T cell subpopulation from young and old donors. **(A)**. PCA for the total CD8^+^ T cell preparations. The variation found was 16% and 28%, respectively. Total CD8^+^ T cells from young donors are blue circles, and old donors are red squares. **(B)**. PCA for the CD8^+^CD45RA^-^ memory T cell subpopulation. The variation found was 12% and 36.5%. The young donors are blue hollow circles and the old donors are red hollow squares.

The PCA plot showed a relatively homogeneous pattern and no clear clustering based on age or sex in the total CD8^+^ T cells with the first two components accounting for 28% and 16%, respectively, of the total variance within the dataset (**Figure 3A** and **Supplementary Figure 3**). In contrast, the PCA plot showed a higher variance within the CD8^+^CD45RA^-^ memory T cell population from old donors, with the first two components accounting for 36.5% and 12%, respectively, of the total variance within the dataset (**Figure 3B**).

### Homogeneity of the Expression Profiles in Total CD8^+^ T Cells, but Increased Heterogeneity in CD8^+^CD45RA^-^ Memory T Cells

**Figure 4:**
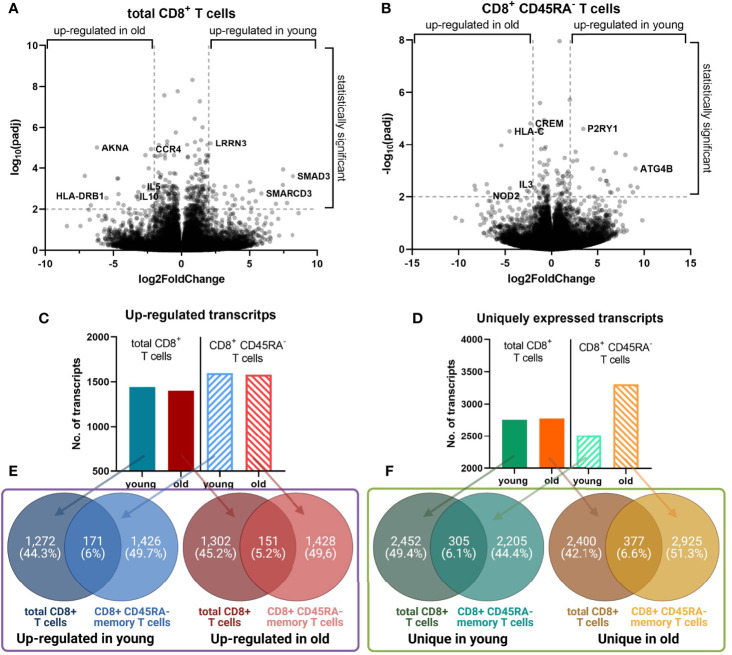
Identified differentially expressed transcripts in young versus old donors. **(A, B)**. Volcano plot for the transcripts with an up-regulated gene expression in the total and memory CD8^+^ T cell populations, respectively. The log_2_(FoldChange) of the transcripts was plotted against the -log_10_(padj). Selected genes with the lowest adjusted p values are labeled. A line is placed at a -log_10_(padj) of 2, which corresponds to an adjusted p value of 0.01. **(C, D)**. The number of transcripts with an up-regulated expression, and uniquely expressed transcripts, respectively. For both CD8^+^ T cell populations the number of transcripts from each dataset are plotted as a graph plot. **(E, F)**. Overlap between the transcripts for the total and memory CD8^+^ T cell population. For both T cell populations, the overlap between the up-regulated and unique transcripts was calculated with Venny 2.1.0 and the figure was prepared with Biorender.com.

Volcano plots demonstrated a similar extent of the log_2_(FoldChange) of the transcript expression for both CD8^+^ T cell populations (**Figures 4A, B**), but total CD8^+^ T cells had an approximately two-fold increase of the number of transcripts with a significant adjusted p value compared to CD8^+^CD45RA^-^ memory T cells with 802 and 442 transcripts, respectively.

The total number of up-regulated transcripts in young and old donors was comparable for both CD8^+^ T cell populations (**Figure 4C**). The number of unique transcripts for total CD8^+^ T cells was almost identical between young and old donors (**Figure 4D**). In contrast, CD8^+^CD45RA^-^ memory T cells of old donors expressed more unique transcripts than those of young donors.

The overlap between the lists of transcripts of the same age, but different cell populations was comparable across the groups (**Figures 4E, F**). For old donors, the overlap of the up-regulated transcripts was 5.5% (151 transcripts), for unique transcripts 6.6% (377 transcripts). Despite the overlap percentage not being significantly different between both age groups, it is noteworthy that the total CD8^+^ T cells of young and old donors are composed of 31 ± 12% and 60 ± 13% CD8^+^CD45RA^-^ memory T cells, respectively (**Figure 2A**).

The distinct transcript expression patterns identified by RNA sequencing were validated by qPCR for selected transcripts chosen based on their stable expression across the donors, which was observed in the RNA sequencing and include CCR4, IL-5, IL-21, SMAD3, LEF1, GATD3A, SLC35A5, U2AF2, ZNF483 for total CD8^+^ T cells and FOXO3, JAK1, IL-3, EZR, GAPVD1, RNF40 and TTC17 for CD8^+^CD45RA^-^ memory T cells demonstrating a comparable expression pattern (**Supplementary Figure 1B**).

### Age-Dependent GO Term Enrichment of Immune Relevant Pathways

**Figure 5:**
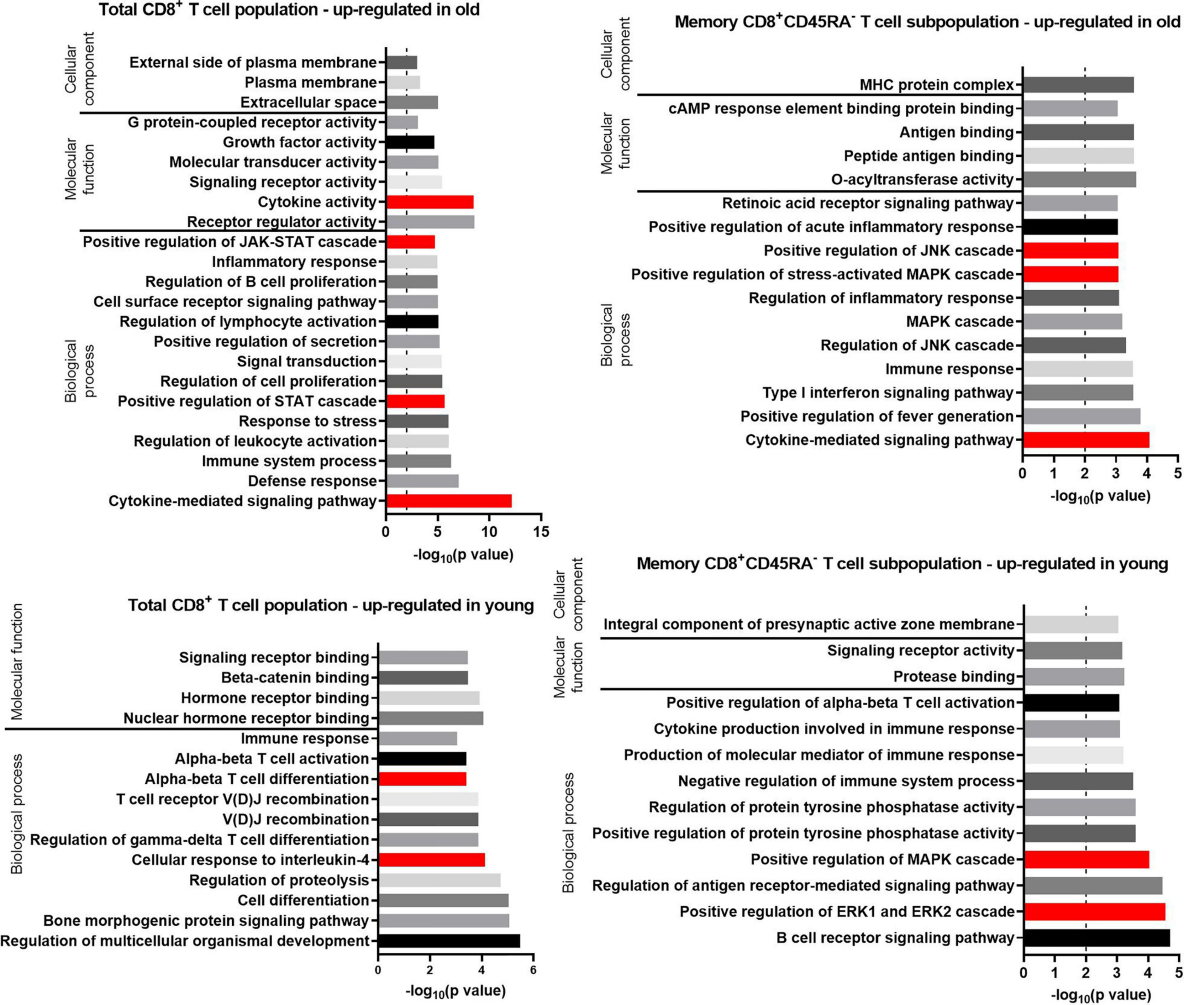
GO terms for transcripts with an up-regulated expression in the total CD8^+^ T cell population and the memory CD8^+^ T cell subpopulation in old and young donors retrieved with GOrilla. The resulting GO terms were classified as biological process, molecular function, or cellular component. The dotted line is indicating −log_10_(p value) = 2, which corresponds to a p value of 0.01.

In the next step, the RNA seq data were analyzed for age-dependent GO enrichment. The number of GO terms and the significance of the interactions were higher for transcripts up-regulated in old donors compared to transcripts up-regulated in young donors (**Figure 5**). For transcripts up-regulated in total CD8^+^ T cells from old donors, the GO term with the lowest p value (7 x 10^-14^) was “cytokine-mediated signaling pathways”, which included transcripts of IL-5, IL-21, IL-10, IL-9, and IL-31. This term was also found for transcripts up-regulated in CD8^+^CD45RA^-^ memory T cells of old donors with a p value of 8 x 10^-5^.

Approximately one third (28.8%) of the biological processes identified for the transcripts up-regulated in total CD8^+^ T cells from old donors referred to immune-related functions or immune-cell activation (**Figure 5**) such as “defense response,” “inflammatory response,” “positive regulation of secretion,” and “regulation of lymphocyte activation.” In addition, several GO terms related to the Janus kinases (JAK) signal transducer and activator of transcription proteins (STAT) pathway and other associated cellular pathways were identified for the transcripts up-regulated in old donors in both total CD8^+^ T cells (5) and CD8^+^CD45RA^-^ memory T cells (8).

GO terms for transcripts up-regulated in old donors in the CD8^+^CD45RA^-^ memory T cells mirror the GO terms enriched for the same age group of total CD8^+^ T cells (e.g., “inflammatory response” in total CD8^+^ T cells and “positive regulation of acute inflammatory response” in CD8^+^CD45RA^-^ memory T cells). Terms linked to the JAK-STAT pathway, such as “positive regulation of the mitogen-activated protein kinases (MAPK) cascade” and “positive regulation of the extracellular signal-regulated kinases 1 (ERK1) and ERK2 cascade” were also identified. Despite this similarity, the transcripts annotated with these terms are different in the two T cell populations.

### In-Depth Analysis for the GO Term Enrichment of Up-Regulated Transcripts

**Figure 6:**
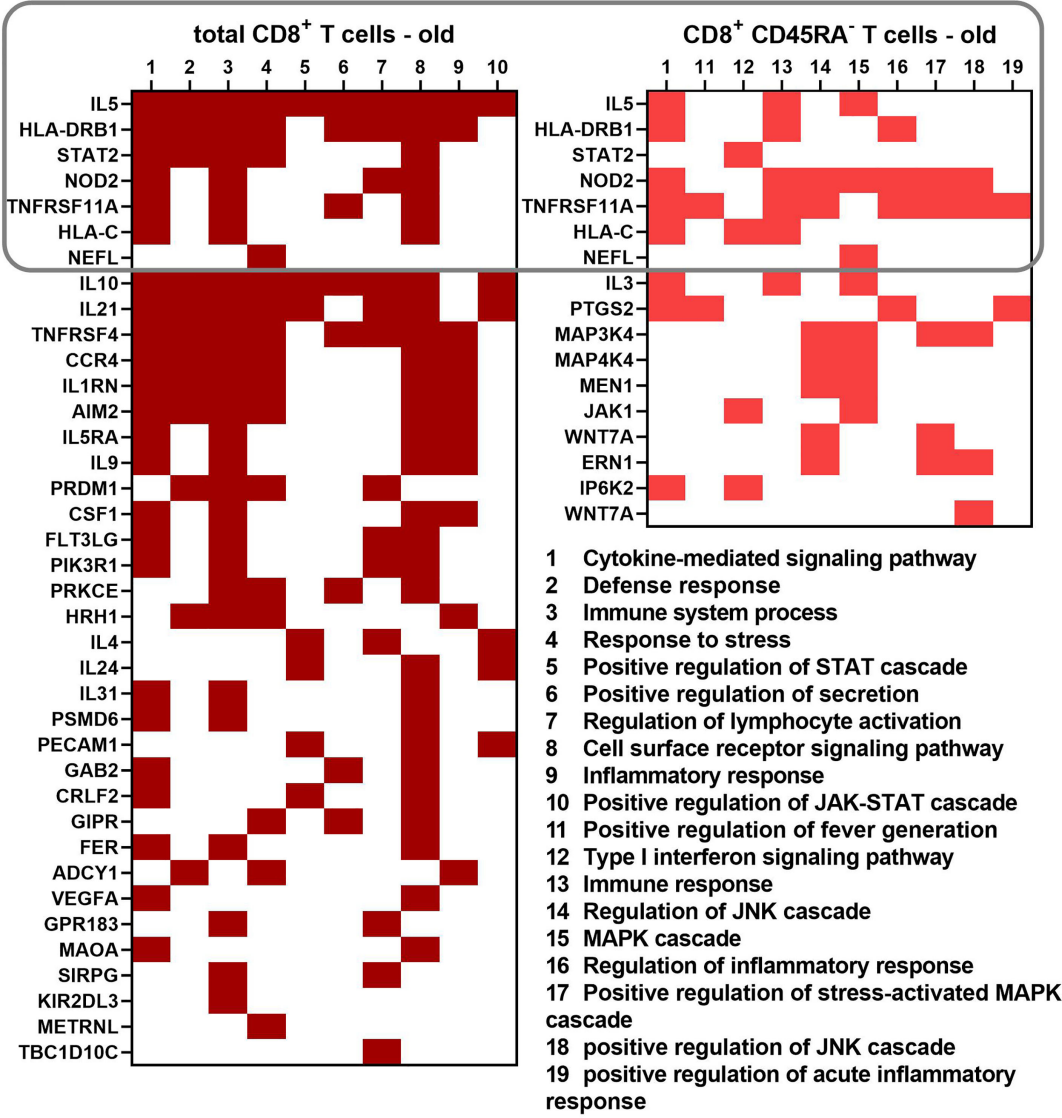
Differentially expressed genes leading to 10 biological process GO terms with the lowest p values for the total and memory CD8^+^ T cell subpopulation of old donors. A binary heatmap (red = yes, white = no) was established using the biological process GO terms with the lowest p values from both old groups. A rectangle indicates the transcripts common for both cell populations.

Genes annotated with the ten most significant GO terms for the biological process category obtained for both CD8^+^ T cell populations of old donors were compared and visualized in a heatmap (**Figure 6**). Transcripts common for both CD8^+^ T cell populations were HLA-C, HLA-DRB1, STAT2, nucleotide-binding oligomerization domain-containing protein 2 (NOD2), IL-5, neurofilament light (NEFL) and TNF receptor superfamily member 11 (TNFRSF11). The number of differentially expressed cytokine transcripts is higher in total CD8^+^ T cells than in CD8^+^CD45RA^-^ memory T cells of old donors with a partial overlap for the term “cytokine-mediated signaling pathway” for both CD8^+^ T cell populations.

IL-5 and HLA-DRB1 were annotated with a high number of the most significant GO terms for total CD8^+^ T cells, whereas both genes were annotated with only three GO terms in CD8^+^CD45RA^-^ memory T cells. A similar pattern was detected for NOD2 and TNFRSF11A, but these genes were more prevalent in CD8^+^CD45RA^-^ memory T cells than in total CD8^+^ T cells.

For the transcripts up-regulated in total CD8^+^ T cells of young donors, 13.5% of the enriched GO terms for Biological Process were associated with cell proliferation and differentiation. GO terms enriched for the CD8^+^CD45RA^-^ memory T cells of young donors were similar to those for total CD8^+^ T cells such as “alpha-beta T-cell activation” in total CD8^+^ T cells and “positive regulation of alpha-beta T-cell activation” in CD8^+^CD45RA^-^ memory T cells. Four terms linked to the JAK-STAT pathway, such as “positive regulation of the MAPK cascade” and “positive regulation of the ERK1 and ERK2 cascade”, were also identified, but the transcripts annotated with these terms were different in T cells of the two age groups.

### GO Term Enrichment Analysis of Uniquely Expressed Transcripts

**Figure 7:**
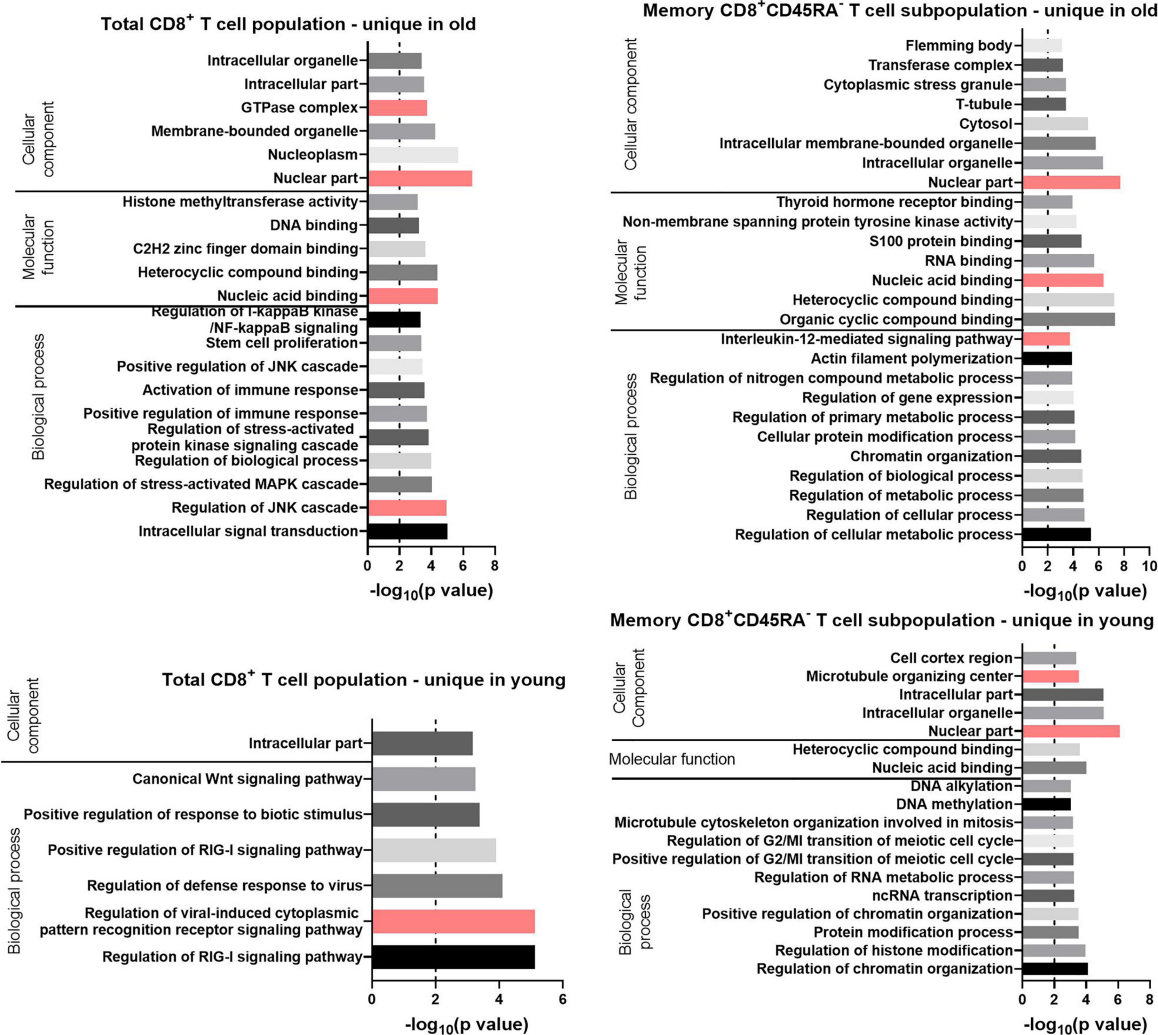
GO terms for the uniquely expressed transcripts retrieved by GOrilla in total CD8^+^ T cells and CD8^+^ CD45RA^-^ memory T cells in old and young donors. All transcripts were ranked according to the normalized mean of the counts for all preparations. The resulting GO terms are classified as biological process, molecular function, or cellular component. The dotted line is indicating −log_10_(p value) = 2, which corresponds to a p value of 0.01.

All of the uniquely expressed transcripts were sorted according to the normalized mean of the counts for all preparations and then processed with GOrilla. CD8^+^CD45RA^-^ memory T cells had a higher number of unique transcripts (**Figure 4D**), so a larger number of GO terms were retrieved by Gorilla for this subpopulation (**Figure 7**).

For the Biological Process category, the GO terms for the total CD8^+^ T cells with the lowest p values were “regulation of the c-Jun N-terminal kinases (JNK) cascade” for the transcripts unique in old (p value = 1 x 10^-5^) and “regulation of viral-induced cytoplasmic pattern recognition receptor signaling pathway” for the transcripts unique in young donors (p value = 7 x 10^-6^). Terms related to the JAK-STAT pathway were found for the transcripts unique in total CD8^+^ T cells of old donors (“regulation of stress-activated MAPK cascade” and “positive regulation of the JNK cascade”). These GO terms were also enriched for the up-regulated transcripts in CD8^+^CD45RA^-^ memory T cells of the same age group. GO terms directly related to the JAK-STAT pathway were not enriched for the transcripts unique in the CD8^+^CD45RA^-^ memory T cells of old donors, but the GO term “interleukin-12-mediated signaling pathway,” a component of the JAK-STAT signaling family, was identified with a p value of 2 x 10^-4^.

For the Molecular Function category, the GO terms “heterocyclic compound binding” and “nucleic acid binding” were retrieved for the unique transcripts in both T cell populations from old donors. For the GO terms enriched in the current lists of transcripts for the Cellular Component category, the term “nuclear part” had a p value under 10^-6^ for all groups except for the total CD8^+^ T cells of the young donors.

## Discussion

Advanced age is associated with alterations in the function of CD8^+^ T cell subpopulations, but the pathways and genes involved in this process have not yet been analyzed in detail, in particular regarding the comparison of total CD8^+^ T cells to memory CD8^+^ T cells. Therefore, this study determined the transcriptional profile in total CD8^+^ T cells and CD8^+^CD45RA^-^ memory T cells from young and old healthy blood donors, to further understand the declined T cell responses against e.g., pathogens and vaccines in the aging population. A hallmark of reduced immunity in old individuals is an increased frequency of CD8^+^CD45RA^-^ memory T cells in the total CD8^+^ T cells (1–7), which was also confirmed by our study (**Figure 2A**). Since total CD8^+^ T cells of young donors have more naïve T cells than that those of old donors, it was hypothesized that the gene expression profiles as well as the function of CD8^+^CD45RA^-^ memory T cells from both age groups are more similar to each other than to those of total CD8^+^ T cells of the corresponding age groups.

Therefore, the age-dependent functionality of both T cell populations was investigated by measuring the surface expression of activation markers (**Figure 2B**) and the concentration of secreted soluble proteins (**Figure 2C**) after 48 h of culture. The discrepancies found for the total CD8^+^ T cells between young and old donors were mirrored by the CD8^+^CD45RA^-^ memory T cells. T cells from old donors had a stronger cytokine secretion and a higher surface expression of CD25 and CD71. The secretion levels of GM-CSF, IL-3, IL-4 and IL-5 were markedly increased in old donors in both cell populations, whereas the soluble factors TNF-α and granzyme A showed no detectable difference between old and young donors. The literature provides evidence of maturation differences in memory T cells in old individuals (6, 47), but little information is available regarding the functionality of these cells compared to the functionality of memory CD8^+^ T cells of young individuals.

Interestingly, the CD8^+^CD45RA^-^ memory T cells mirrored the total CD8^+^ T cells of the same age group at the transcriptional and functional level despite the different composition of both T cell populations in young and old donors (**Figure 2A**). Other studies made a similar observation by demonstrating that CD8^+^CD28^+^ T cells of old donors are more similar to CD8^+^CD28^-^ T cells of the same age group than to the same subpopulation of a different age group (26). CD28^-^ T cells exhibit a weak immune responsiveness in addition to shorten telomeres (48). A significant difference was observed in the number of uniquely expressed transcripts, which was increased for CD8^+^CD45RA^-^ memory T cells from old donors (**Figure 4D**) suggesting that the gene expression profile of this subpopulation is different than the other groups. However, the overlap in transcripts was similar when comparing the transcript list of the same age group, but different cell populations (**Figures 4E, F**).

To obtain further insights into the relevance of the observed differential gene expression patterns, the functional profile of each list of transcripts was acquired with GO term enrichment analyses. For transcripts up-regulated in total CD8^+^ T cells of old donors, the JAK-STAT pathway and the “cytokine-mediate signaling pathway” had the most significant p values. The JAK-STAT pathway is involved in the signal transduction of multiple cytokines including IL-2 (34), IL-5 (49, 50), IL-4 (51), IFN-γ (36) and other soluble factors (52, 53). Thus, the JAK-STAT pathway regulates the activation, proliferation and cytotoxicity of T cells, while there is evidence that defective signaling within this pathway results in chronic inflammation (53, 54) or premature aging of stem cells (55). In addition, JAK inhibitors, such as the JAK1/2 inhibitor ruxolitinib, alleviate premature aging effects for the Hutchinson-Gilford progeria syndrome (56). In addition, increased cytokine production in T cells of old individuals was reported (21, 22, 57, 58). In this study, a correlation between the increased cytokine secretion and activation was identified in CD8^+^ T cells of old donors upon examining the soluble proteins in the supernatant, the surface expression of activation markers and the presence of the JAK-STAT pathway in the set of enriched GO terms in CD8^+^ T cells from old donors.

For transcripts up-regulated in CD8^+^CD45RA^-^ memory T cells from old donors several enriched GO terms were associated with the JAK-STAT cascade such as “positive regulation of the MAPK cascade” and “positive regulation of ERK1 and ERK2 cascade.” Multiple kinases from the MAPK pathway were shown to activate the JAK-STAT pathway including ERK (59) and JNK (60), which is known to phosphorylate STAT1 and STAT3 upon inhibition of the MAPK pathway (59). Both signaling pathways are activated by the same ligands (61) and also modulate the expression of a number of similar genes and are known to coordinate senescence and inflammation (62–64).

Additional GO terms enriched in the set of transcripts up-regulated in CD8^+^CD45RA^-^ memory T cells of old donors related to the “cytokine-mediate signaling pathways” such as “positive regulation of fever generation.” Fever represents a consequence of inflammation, which is a key element of the immune response (65). In a murine model, it was shown that naïve CD8^+^ T cells differentiate into CD62L^lo^ CD44^hi^ effector T cells in a temperature-dependent manner both *in vivo* and *in vitro* (66).

Despite the apparent overlap of the different GO terms enriched in the sets of transcripts up-regulated in the two CD8^+^ T cell populations of old donors, the only common transcripts were IL-5, HLA-DRB1, HLA-C, STAT2, NOD2, TNFRSF11A, and NEFL (**Figure 6**). TNFRSF11A, IL-5, and other cytokines directly bind to several JAK and STAT proteins (67, 68). HLA-C was found up-regulated in CD8^+^ T cells from the cerebrospinal fluid of Alzheimer’s patients through RNA sequencing (69). Interestingly, a subpopulation of HLA-DR^+^CD8^+^ T cells was described, which has regulatory properties mediated by direct cell contact (15, 16). HLA-DRB1 was used as a biomarker for dry eye diseases, which correlated with an increase in pro-inflammatory and JAK-STAT related genes (70). Our study suggests an up-regulated frequency in HLA-DRB1^+^ CD8^+^ T cells in old blood donors, which is in line with the increased number of CD8^+^HLA-DR^+^ T cells during aging next to other CD8^+^ T cell memory phenotypes (17). However, the role of this T subpopulation during the aging process has not yet been demonstrated and thus deserves further investigation.

A large number of transcripts was found to be uniquely expressed in one of the age groups with the highest number of such transcripts in CD8^+^CD45RA^-^ memory T cells of old donors (**Figure 4D**). The term “interleukin-12-mediated signaling pathway” was enriched for transcripts uniquely expressed in CD8^+^CD45RA^-^ memory T cells of old donors. IL-12 signaling is associated with the JAK-STAT pathway (60) and has been shown to trigger IFN-γ secretion by activating the STAT4 protein (68, 71).

In contrast to these observations, several studies demonstrated that this pathway was defective in old individuals (54, 72), but a comparison of the expression of this pathway in memory CD8^+^ T cells of young versus old donors was not performed in these studies. When comparing the transcripts expression profiles of different CD8^+^ T cells populations, the JAK-STAT pathway and related pathways were present both in up-regulated transcripts and in uniquely expressed transcripts in CD8^+^ T cells from old donors both in total CD8^+^ T cells and CD8^+^CD45RA^-^ memory T cells.

Overall, our results suggest an altered expression of cytokine pathways in total and memory CD8^+^ T cells of old individuals when compared to that of young donors. Further studies are required to confirm the regulatory function of the JAK/STAT pathway, which might give insights into how to revert these expression changes in T cells of old donors.

## Conclusions

While many studies have shown that the distribution of various CD8^+^ T cell subpopulations changes with aging, few comparisons of different T cell subpopulations were made within the same age group. In this study, the transcriptional profile, secretion, and surface marker expression of CD8^+^CD45RA^-^ memory T cells of young and old donors were analyzed and compared to those of total CD8^+^ T cells of young and old donors. Our results demonstrate that the secretion patterns of both total and memory CD8^+^ T cells were higher in old individuals than in young individuals, which correlated to the substantial changes in the gene expression during the aging process.

In addition, both T cell populations showed an age-dependent increase in CD25 surface expression, indicating an increased potential for T cell activation in the old individuals. Functional annotation analyses indicate that aging affects transcripts involved in the JAK-STAT pathway with an increased expression in total CD8^+^ T cells and CD8^+^CD45RA^-^ memory T cells of old donors. Moreover, pathways related to the JAK-STAT cascade were enriched for transcripts uniquely expressed in old donors both for total CD8^+^ T cells and for the CD8^+^CD45RA^-^ memory subpopulation.

Ultimately, CD8^+^CD45RA^-^ memory T cells of old donors had a functional and transcriptional profile comparable to total CD8^+^ T cells from old donors rather than CD8^+^CD45RA^-^ memory T cells from young donors. Thus, both total and memory CD8^+^ T cells undergo an age-related functional decline, which is reflected by changes in specific genes and signaling pathways. These might provide attractive targets for future studies on T cell aging.

## Data Availability Statement

The datasets presented in this study can be found in online repositories. The names of the repository/repositories and accession number(s) can be found below: NCBI SRA BioProject, accession no: PRJNA790638.

## Author Contributions

GT: experimental design, physical lab work, writing (first draft), writing (editing and review), figure preparation. IL: experimental design, statistical analysis, writing (first draft), writing (editing and review), figure preparation. EK: additional lab work, validation. IG: conceptualization, writing (editing and review). BS: conceptualization, writing (editing and review). All authors: contributed to the article and approved the submitted version.

## Funding

This study was funded by the German Research Foundation (ProMoAge, grant no. GRK 2155, subproject 5, BS). And we acknowledge the financial support of the Open Access Publication Fund of the Martin-Luther-University Halle-Wittenberg.

## Conflict of Interest

The authors declare that the research was conducted in the absence of any commercial or financial relationships that could be construed as a potential conflict of interest.

## Publisher’s Note

All claims expressed in this article are solely those of the authors and do not necessarily represent those of their affiliated organizations, or those of the publisher, the editors and the reviewers. Any product that may be evaluated in this article, or claim that may be made by its manufacturer, is not guaranteed or endorsed by the publisher.

## Footnotes

1
https://www.bioinformatics.babraham.ac.uk/projects/fastqc/

2
https://www.r-project.org/

3
http://cbl-gorilla.cs.technion.ac.il/

4
https://bioinfogp.cnb.csic.es/tools/venny/index.html

5
https://biorender.com/

## Supplementary Material

Supplementary Figure 1 Flow cytometry gating strategy. The first gate of forward scatter (FSC) versus side scatter (SSC) was used to discriminate the cells from possible debris or bubbles in the machine. The second gate set on PE and PerCP-Cy5.5 distinguished between live and dead cells; the double-negative cells in this gate were the living cells. The third gate was used for doublet exclusion by plotting the FSC area against its width. Once the live single lymphocytes were determined, these cells could be further separated based on their surface markers. The next gate was set on FITC versus Qdot 605 (same as BV605), which allowed for the evaluation of CD3 and CD8, respectively. The CD3^+^CD8^+^ T cells were further gated for APC versus Qdot 6005, representing CD45RA versus CD8, respectively. This final gate was used only for the preparation of the memory CD8^+^ CD45RA^-^ T cells.

Supplementary Figure 2 Validation of RNA seq data. **(A)**. Relative gene expression of cytokines after 48h of activation. Total RNA was isolated from total and memory CD8^+^ T cells and qPCRs were prepared for the selected cytokines. SDHA and TBP served as housekeepers. The ΔΔCq value was calculated using the Cq values for the young versus those for the old donors. Therefore, a negative value represents an increase in the expression of the respective gene in the old donors and a positive value an increase in the young. N = 8. **(B)**. Comparison of the qPCR and RNA seq values for selected genes. qPCRs were prepared for the selected genes, using SDHA and TBP as housekeepers. The ΔΔCq value was calculated using the young versus the old donors, so that the change in expression can be compared to RNA seq log_2_(FoldChange). N = 8.

Supplementary Figure 3 Principal Component Analysis of the transcriptomic profiles of the total CD8^+^ T cell population and the CD8^+^CD45RA^-^ memory T cell subpopulation from young and old donors divided by gender. **(A)**. PCA for the total CD8^+^ T cell preparations. Total CD8^+^ T cells from young female donors are light red squares, young male donors light blue squares, old female donors dark red squares, and old male donors dark blue squares. **(B)**. PCA for the CD8^+^CD45RA^-^ memory T cell subpopulation. The young female donors are light red hollow circles, young male donors light blue hollow circles, old female donors dark red hollow circles and old male donors dark blue hollow circles.

## Abbreviations

cDNA, complementary DNA; Cq, quantification cycle; ERK, extracellular signal-regulated kinases; GM-CSF, granulocyte-macrophage colony-stimulating factor; GO, gene ontology; GOrilla, gene ontology enrichment analysis and visualization tool; IFN-γ, interferon-gamma; IL, interleukin; JAK, Janus kinases; JNK, c-Jun N-terminal kinases; mAb, monoclonal antibody; MAPK, mitogen-activated protein kinases; NEFL, neurofilament light; NOD2, nucleotide-binding oligomerization domain-containing protein 2; PBMC, peripheral blood mononuclear cells; PBS, phosphate-buffered saline; PCA, principal component analysis; qPCR, quantitative PCR; SDHA, succinyl dehydrogenase subunit A; STAT, signal transducer and activator of transcription proteins; TBP, TATA-binding box protein; TNF-α, tumor necrosis factor-alpha; TNFRSF11, TNF receptor superfamily member 11.
