# Supplementary material for: Transcriptional Analysis of Total CD8+ T Cells and CD8+CD45RA- Memory T Cells From Young and Old Healthy Blood Donors
Source: Front Immunol. 2022 Jan 27;13:806906. doi: 10.3389/fimmu.2022.806906 (PMC8829550; doi:10.3389/fimmu.2022.806906)
Supplement: Supplementary file 1 [file Table_1.docx]

Supplementary table 1. Donor information for all the preparations for RNA sequencing. Young (Y) and old donors (O) are grouped based on the CD8^+^ T cell populations. For each donor, the age, sex, body mass index (BMI) and cytomegalovirus (CMV) infection status. The mean and standard deviation (stdv) are calculated when possible.

|  | Donor | Age | Sex | BMI | CMV status |
| --- | --- | --- | --- | --- | --- |
| Total CD8^+^ T cell population | Y11 | 29 | M | 30.42 | - |
|  | Y12 | 27 | M | 27.70 | - |
|  | Y13 | 27 | M | 26.56 | - |
|  | Y14 | 23 | F | 19.53 | + |
|  | Y15 | 27 | F | 33.41 | - |
|  | Y16 | 24 | M | 34.54 | + |
|  | Y17 | 25 | F | 22.60 | + |
|  | Y18 | 23 | F | 19.84 | - |
|  | Mean ± Stdv | 25.63 ± 2.19 |  | 26.82 ± 5.81 |  |
|  | O11 | 61 | M | 27.77 | + |
|  | O12 | 62 | F | 25.99 | + |
|  | O13 | 65 | F | 27.01 | + |
|  | O14 | 61 | M | 31.83 | + |
|  | O15 | 64 | M | 27.76 | + |
|  | O16 | 68 | M | 27.77 | - |
|  | O17 | 65 | F | 43.60 | - |
|  | O18 | 66 | F | 26.35 | - |
|  | Mean ± Stdv | 64 ± 2.5 |  | 29.76 ± 5.87 |  |
| CD8^+^CD45RA^-^ memory  T cell population | Y22 | 28 | F | 17.56 | + |
|  | Y23 | 27 | F | 33.41 | - |
|  | Y24 | 24 | M | 20.30 | - |
|  | Y25 | 30 | M | 25.25 | - |
|  | Y26 | 25 | F | 22.60 | + |
|  | Y27 | 25 | M | 20.91 | - |
|  | Y28 | 23 | F | 20.91 | - |
|  | Mean | 26 ± 2.44 |  | 22.99 ± 4.77 |  |
|  | O21 | 64 | M | 27.76 | + |
|  | O22 | 60 | F | 22.55 | - |
|  | O23 | 63 | M | 27.40 | + |
|  | O24 | 68 | M | 27.77 | - |
|  | O25 | 64 | M | 33.24 | - |
|  | O26 | 66 | F | 26.35 | - |
|  | O27 | 62 | F | 34.48 | + |
|  | O28 | 64 | F | 26.22 | + |
|  | Mean | 63.88 ± 2.41 |  | 28.22 ± 3.87 |  |

Supplementary table 2. Primers for validation of RNA sequencing data. All primers used for qPCR were purchased from Integrated DNA Technologies (Coralville, Iowa, U.S.A.) and dissolved in Ampuwa water to a final concentration of 10 mM (Fw = forward, 5’–3’; Rw = reverse, 3’–5’).

| Gene | Transcript | Sense | Sequence 5'→3' | Product length | Annealing temperature |
| --- | --- | --- | --- | --- | --- |
| *Granzyme B (Morissette, 2007)* | NM_001346011.2 | Fw | TGGGGGACCCAGAGATTAAAA | 100 | 58 |
|  |  | Rw | TTTCGTCCATAGGAGACAATGC |  |  |
| *Granzyme A (Hochegger, 2007)* | NM_006144.4 | Fw | TTTCTGGCATCCTCTCTCTCA | 305 | 58 |
|  |  | Rw | GGGTCATAGCATGGATAGGG |  |  |
| *TNF-a* | NM_000594.4 | Fw | GCTGCACTTTGGAGTGATCG | 215 | 56 |
|  |  | Rw | TATCTCTCAGCTCCACGCCA |  |  |
| *IFN-γ* | NM_000619.3 | Fw | GCTCTGCATCGTTTTGGGTT | 488 | 56 |
|  |  | Rw | ATTGCAGGCAGGACAACCAT |  |  |
| *IL-10 (Vigano, 2001)* | NM_000572.3 | Fw | CTGTGAAAACAAGAGCAAGGC | 500 | 58 |
|  |  | Rw | GAAGCTTCTGTTGGCTCCC |  |  |
| *IL-6* | NM_000600.5 | Fw | TTCGGTCCAGTTGCCTTCTC | 316 | 56 |
|  |  | Rw | TCACCAGGCAAGTCTCCTCA |  |  |
| *IL-5* | NM_000879.3 | Fw | AGCCAATGAGACTCTGAGGAT | 359 | 56 |
|  |  | Rw | AGGCCTGACTCTTTCTTGGC |  |  |
| *IL-4* | NM_000589.4 | Fw | TCTTTGCTGCCTCCAAGAACA | 235 | 56 |
|  |  | Rw | TCCAACGTACTCTGGTTGGC |  |  |
| *IL-3* | NM_000588.4 | Fw | CCTTTGCCTTTGCTGGACTTC | 335 | 56 |
|  |  | Rw | AGAGAACGAGCTGGACGTTG |  |  |
| *CCR4* | XM_017005687.1 | Fw | GGCTTACACCCACAGTGGAA | 307 | 60 |
|  |  | Rw | ATTAGAGGGCCCATTTGCCA |  |  |
| *IL-21* | NM_021803.4 | Fw | ACCTTCCACAAATGCAGGGA | 497 | 58 |
|  |  | Rw | CTGGTGGTAATCAAGCTCAAGG |  |  |
| *SMAD3* | NM_001145104.1 | Fw | GGGAAAAATCGATGAGCGCC | 414 | 60 |
|  |  | Rw | AGAAGCAGTTGCTGGGTCTC |  |  |
| *LEF1* | XM_005263047.1 | Fw | AACTTGGCTGCATTTGCCTG | 394 | 60 |
|  |  | Rw | GAACACCTTACAAGGGCGGA |  |  |
| *GATD3A* | NM_004649.8 | Fw | GAAGGCGAGAGCAGGAATGT | 197 | 60 |
|  |  | Rw | TTCAGGACACGCTCCACTTC |  |  |
| *SLC35A5* | NM_001348906.1 | Fw | CGCACCTAGGCAAGAAAGGA | 418 | 60 |
|  |  | Rw | GGAAGCTTGAAGGCCAAGGA |  |  |
| *U2AF2* | XM_011526410.1 | Fw | GTACAAGGCCATGCAAGCTG | 299 | 60 |
|  |  | Rw | CCACTGAGCGGAACTCCAAA |  |  |
| *ZNF483* | XM_017014337.1 | Fw | AATTCATGCCTTACCCGGCA | 461 | 60 |
|  |  | Rw | TCTGCAGAGTGAAATCCCCG |  |  |
| *FOXO3* | XM_011535629.2 | Fw | GCCACCCTTGGCCTCTAAAT | 463 | 60 |
|  |  | Rw | CGGGGCATACTTGTCCTGAG |  |  |
| *JAK1* | NM_001321856.1 | Fw | ATCTGCACAGAAGACGGAGG | 371 | 60 |
|  |  | Rw | GACCAGACGTCAGAGGCAAT |  |  |
| *IL7RE* | XR_940382.3 | Fw | ACAGCCAGCACACTCAGATG | 500 | 60 |
|  |  | Rw | AAGAAGGCTTCCCCAGTCAC |  |  |
| *EZR* | XM_011536110.1 | Fw | CGGGACAAGTACAAGACGCT | 152 | 60 |
|  |  | Rw | CTAAAGACACAAGCGTGGCG |  |  |
| *TTC17* | XM_006718263.1 | Fw | GTGAGTACCTGGCTTGCAGT | 298 | 60 |
|  |  | Rw | CCCAGGACGTCTGGTTCTTT |  |  |
| *GAPVD1* | XM_024447503.1 | Fw | GCAGAAGCCGCACCAATATG | 232 | 59 |
|  |  | Rw | CGATTTGAAGGACCATGGAGT |  |  |
| *RNF40* | NM_001286572.3 | Fw | AAGAGGGCTCAGGAGGACAT | 477 | 60 |
|  |  | Rw | TGGAACGAAGCTTGGCTGAT |  |  |
