# Supplementary figures and images for: Transcriptional Analysis of Total CD8+ T Cells and CD8+CD45RA- Memory T Cells From Young and Old Healthy Blood Donors
Source: Front Immunol. 2022 Jan 27;13:806906. doi: 10.3389/fimmu.2022.806906 (PMC8829550; doi:10.3389/fimmu.2022.806906)

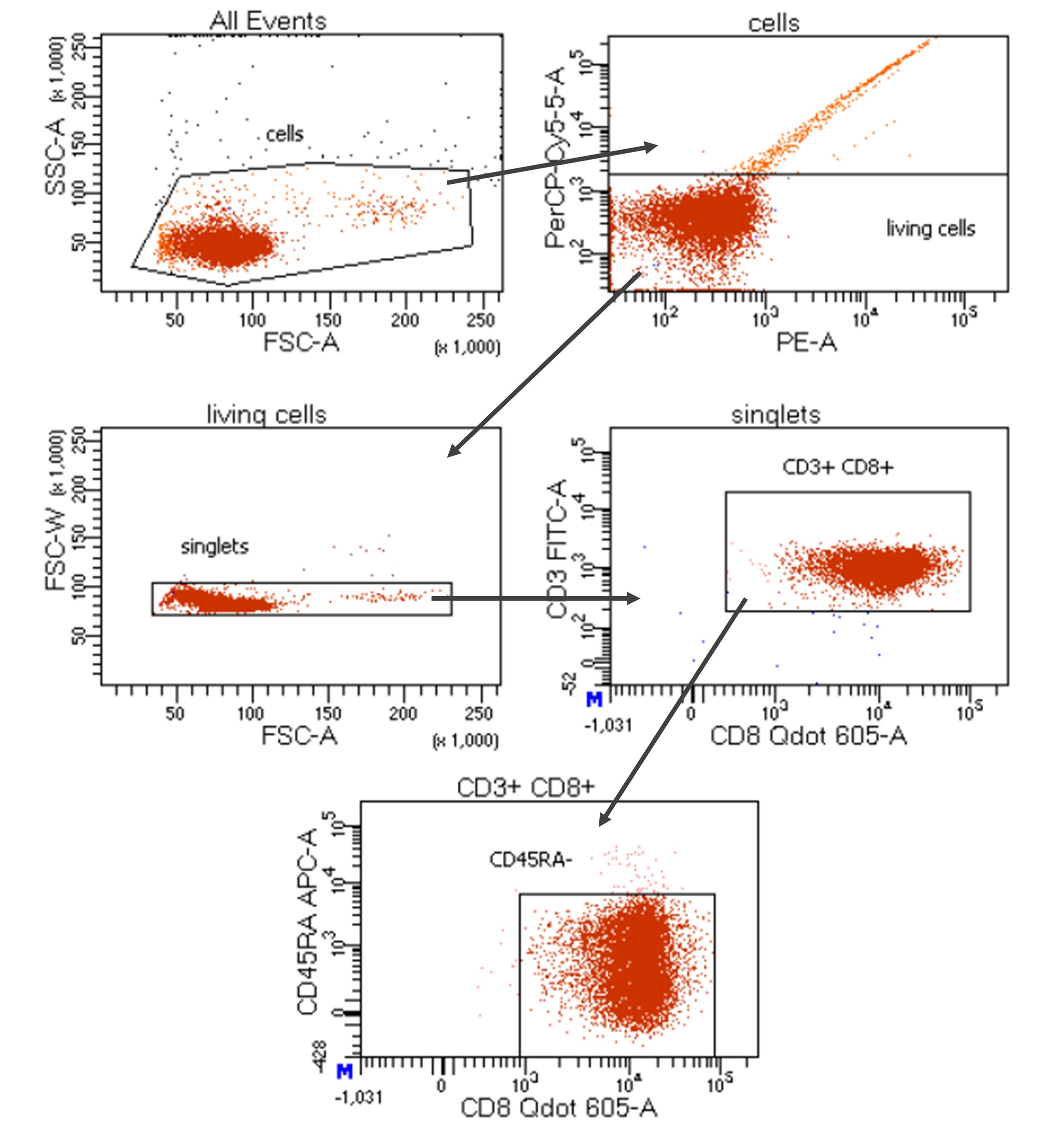

Supplement: Supplementary Figure 1 — Flow cytometry gating strategy. The first gate of forward scatter (FSC) versus side scatter (SSC) was used to discriminate the cells from possible debris or bubbles in the machine. The second gate set on PE and PerCP-Cy5.5 distinguished between live and dead cells; the double-negative cells in this gate were the living cells. The third gate was used for doublet exclusion by plotting the FSC area against its width. Once the live single lymphocytes were determined, these cells could be further separated based on their surface markers. The next gate was set on FITC versus Qdot 605 (same as BV605), which allowed for the evaluation of CD3 and CD8, respectively. The CD3+CD8+ T cells were further gated for APC versus Qdot 6005, representing CD45RA versus CD8, respectively. This final gate was used only for the preparation of the memory CD8+ CD45RA- T cells. [file Image_1.jpeg]

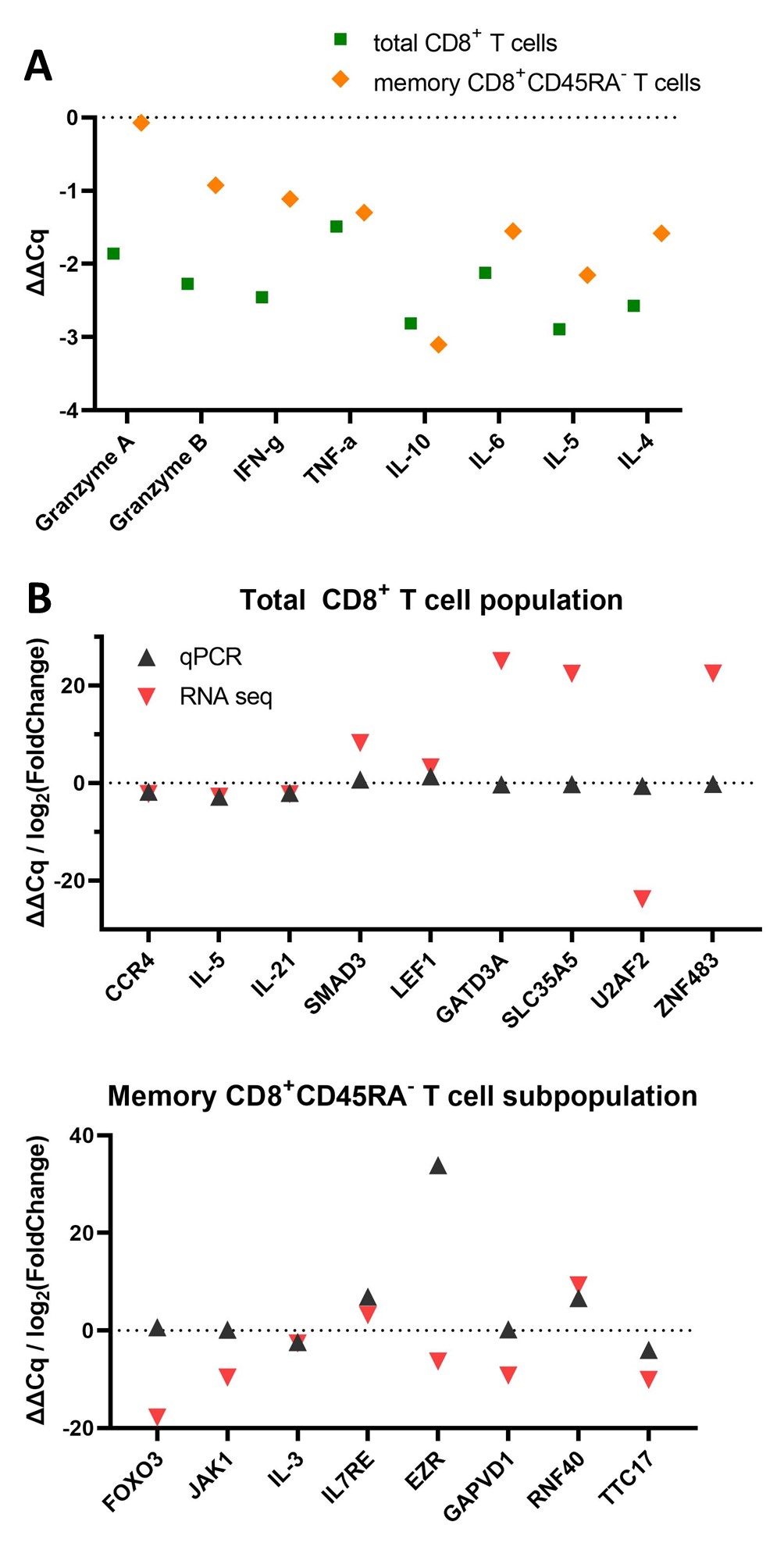

Supplement: Supplementary Figure 2 — Validation of RNA seq data. (A). Relative gene expression of cytokines after 48h of activation. Total RNA was isolated from total and memory CD8+ T cells and qPCRs were prepared for the selected cytokines. SDHA and TBP served as housekeepers. The ΔΔCq value was calculated using the Cq values for the young versus those for the old donors. Therefore, a negative value represents an increase in the expression of the respective gene in the old donors and a positive value an increase in the young. N = 8. (B). Comparison of the qPCR and RNA seq values for selected genes. qPCRs were prepared for the selected genes, using SDHA and TBP as housekeepers. The ΔΔCq value was calculated using the young versus the old donors, so that the change in expression can be compared to RNA seq log2(FoldChange). N = 8. [file Image_2.jpeg]

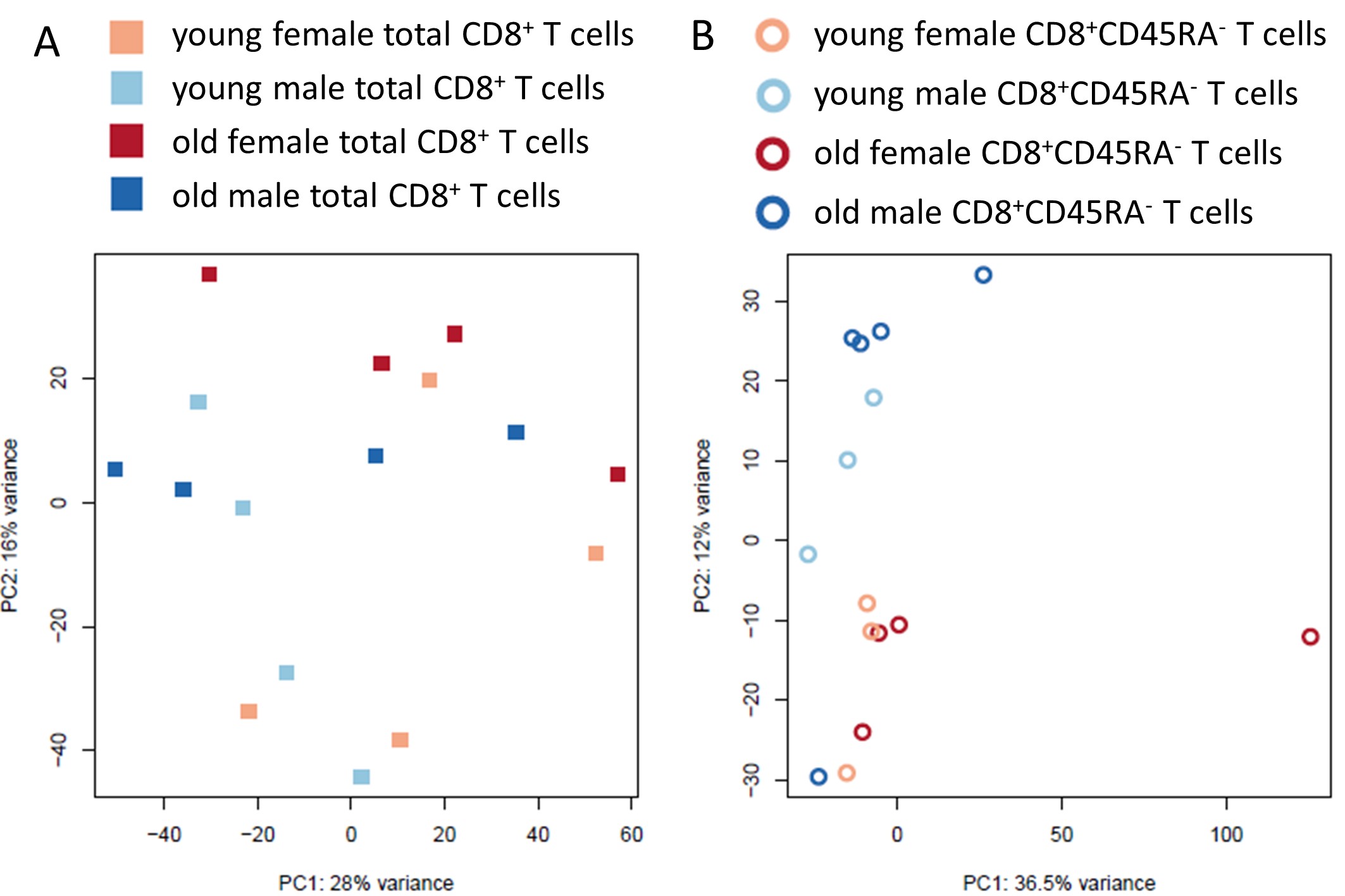

Supplement: Supplementary Figure 3 — Principal Component Analysis of the transcriptomic profiles of the total CD8+ T cell population and the CD8+CD45RA- memory T cell subpopulation from young and old donors divided by gender. (A). PCA for the total CD8+ T cell preparations. Total CD8+ T cells from young female donors are light red squares, young male donors light blue squares, old female donors dark red squares, and old male donors dark blue squares. (B). PCA for the CD8+CD45RA- memory T cell subpopulation. The young female donors are light red hollow circles, young male donors light blue hollow circles, old female donors dark red hollow circles and old male donors dark blue hollow circles. [file Image_3.jpeg]
